# A prospective, multicenter, open-label, single-arm clinical trial design to evaluate the safety and efficacy of ^90^Y resin microspheres for the treatment of unresectable HCC: the DOORwaY90 (Duration Of Objective Response with arterial Ytrrium-90) study

**DOI:** 10.1186/s12876-022-02204-1

**Published:** 2022-03-28

**Authors:** Armeen Mahvash, Steven Chartier, Mark Turco, Paula Habib, Steven Griffith, Scott Brown, S. Cheenu Kappadath

**Affiliations:** 1 Department of Interventional Radiology, Unit 1471, University of Texas MD Anderson Cancer Center, 1515 Holcombe Boulevard, Houston, TX 77030 USA; 2 Sirtex, Woburn, MA USA; 3 BRIGHT Research Partners, Minneapolis, MN USA; 4 Department of Imaging Physics, University of Texas MD Anderson Cancer Center, 1155 Pressler Street, Unit 1352, Houston, TX 77030 USA

**Keywords:** Radioembolization, SIRT, ^90^Y resin microspheres, Hepatocellular carcinoma (HCC)

## Abstract

**Background:**

Selective internal radiation therapy (SIRT) with yttrium-90 (^90^Y) resin microspheres is an established locoregional treatment option for unresectable hepatocellular carcinoma (HCC), which delivers a lethal dose of radiation to hepatic tumors, while sparing surrounding healthy tissue. DOORwaY90 is a prospective, multicenter, open-label, single arm study, designed to evaluate the safety and effectiveness of ^90^Y resin microspheres as first-line treatment in patients with unresectable/unablatable HCC. It is unique in that it is the first study with resin microspheres to utilize a personalized ^90^Y dosimetry approach, and independent review for treatment planning and response assessment.

**Methods:**

Eligibility criteria include unresectable/unablatable HCC, Barcelona Clinic Liver Cancer stage A, B1, B2, or C with a maximal single tumor diameter of ≤ 8 cm, and a sum of maximal tumor diameters of ≤ 12 cm, and at least one tumor ≥ 2 cm (long axis) per localized, modified Response Evaluation Criteria in Solid Tumors. Partition model dosimetry is used to determine the optimal dose; the target mean dose to tumor is ≥ 150 Gy. Patients are assessed at baseline and at regular intervals up until 12 months of treatment for response rates, safety, and quality of life (QoL). Post-treatment dosimetry is used to assess dose delivered to tumor and consider if retreatment is necessary. The co-primary endpoints are best objective response rate and duration of response. Secondary endpoints include grade ≥ 3 toxicity, QoL, and incidence of liver resection and transplantation post SIRT. Target recruitment is 100 patients.

**Discussion:**

The results of this trial should provide further information on the potential use of SIRT with ^90^Y resin microspheres as first-line therapy for unresectable HCC.

***Trial registration*:**

Clinicaltrials.gov; NCT04736121; date of 1st registration, January 27, 2021, https://clinicaltrials.gov/ct2/show/NCT04736121.

**Supplementary Information:**

The online version contains supplementary material available at 10.1186/s12876-022-02204-1.

## Background

Hepatocellular carcinoma (HCC) is the most common type of primary liver cancer, with the third highest solid tumor mortality rate after lung and colon cancer [1]. In the USA, it is estimated that in 2020, 42,810 new cases of HCC were diagnosed, with a 5-year survival rate of 18% [2]. In early-intermediate HCC, liver resection or liver transplantation may be appropriate treatment options [3], but approximately 70–80% of patients with HCC are not diagnosed early enough to benefit from these potentially curative treatments [4, 5].

Several treatment strategies are available for patients with unresectable HCC. One such strategy is selective internal radiation therapy (SIRT), also referred to as radioembolization. SIRT is a technique that selectively deposits yttrium-90 (^90^Y) microspheres into the hepatic vasculature to deliver a lethal dose of radiation to the tumor(s). Surrounding healthy liver tissue can be spared by selectively delivering the microspheres into specific Couinaud segment(s) or the tumor vasculature itself. SIRT exploits the tumor’s hypervascularity relative to healthy tissue, delivering safe levels of radiation to normal tissue while achieving a selective tumoricidal dose. Relative to bland embolization or chemoembolization, SIRT minimizes potential macroembolic effect. Instead, SIRT leads to partial occlusion (microembolization) of the vascular supply to the tumor while the beta-radiation emitted from ^90^Y induces DNA damage in tumor cells [6, 7].

SIRT has the potential to not only delay local progression but also to downstage the disease to allow resection or transplantation if the disease meets Milan, or similar, criteria [8–10]. Previous randomized clinical trials of first-line ^90^Y resin microspheres (SIR-Spheres®, Sirtex Medical) for advanced HCC did not meet their primary endpoints for a number of reasons that include: (1) inadequate dosimetry-treatment planning; (2) poorly controlled patient populations; and (3) no post-treatment confirmation of absorbed dose [11–13]. These trials were initiated and conducted before the development of our current understanding of radiation-dose–response following SIRT, and before the widespread acceptance of personalized dosimetry for SIRT [14].

To overcome the limitations of these trials, we initiated the Duration Of Objective Response with arterial Yttrium-90 (DOORwaY90) study of ^90^Y resin microspheres, which utilizes a personalized dosimetry approach using the partition model and is recruiting patients with unresectable HCC (Barcelona Clinic Liver Cancer [BCLC] stage A, B1, B2 and C). All eligible patients receive ^90^Y resin microspheres using either a selective or lobar approach.

## Methods/design

### Study design

The DOORwaY90 trial is a pivotal, prospective, multicenter, open-label, single-arm study. The objective is to evaluate the safety and effectiveness of ^90^Y resin microspheres as first-line treatment for unresectable HCC (BCLC stages A, B1, B2, and C).

**Fig. 1:**
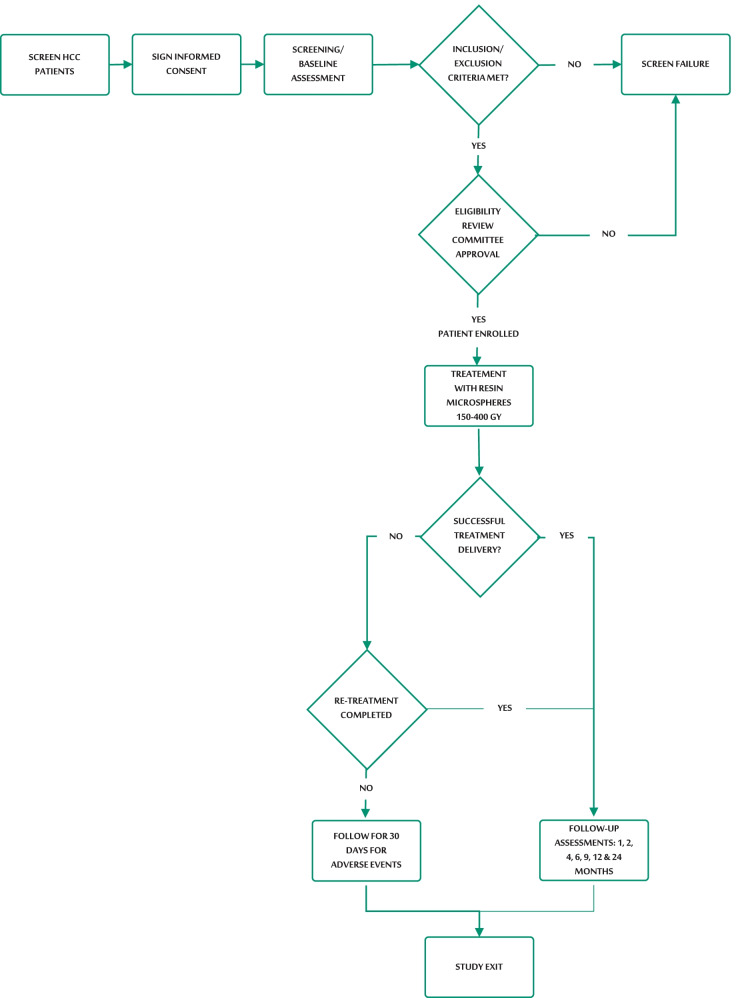
Study flow diagram

All relevant institutional and local ethics committee approvals were gained before commencing the study (Additional file 1). The study is being conducted in 14 investigational sites in the USA and aims to enroll approximately 100 patients (Fig. 1). No single site will enroll more than 20% of the study population. Written informed consent is obtained from all participants (or their legally authorized representatives) prior to participation in this study.

Investigators and study sites are required to permit study-related monitoring, audits, Institutional Review Board (IRB) review, and regulatory inspection(s) and provide direct access to source data/documents. This study will have oversight by a Data and Safety Monitoring Board (DSMB), which will review summarized safety data (including enrollment, protocol deviations, and adverse events) and will use stopping rules as defined in the DSMB Charter. The sponsor may also terminate or suspend the study if the DSMB stopping rules have been met. Sites must submit any protocol amendments to the IRB and are required to forward a copy of the written approval to the CRO. The final dataset will be available to the sponsor/CRO and IRB.

Important aspects of the trial include: (1) personalized dosimetry for treatment planning; (2) centralized review of the treatment plans and verification scans; (3) use of an independent core laboratory for response assessment; (4) a specific patient population that would benefit from locoregional treatment; (5) potentially curative treatment; (6) confirmation of absorbed dose delivered to the tumor; and (7) assessment of long-term toxicity.

### Patients

Adult (≥ 18 years of age) patients enrolled into this study have a diagnosis of HCC BCLC stage A, B1, B2, or C with a maximal single lesion size of ≤ 8 cm and a sum of maximal tumor diameters of ≤ 12 cm, with the entire tumor burden expected to be treatable within the perfused volume. Patients cannot be considered suitable for treatment by resection or ablation at the time of study entry. For safety considerations, ≥ 33% of the total liver volume (body surface area-based) must be disease free and not treated with ^90^Y [15]. All inclusion and exclusion criteria are summarized in Table 1.

**Table 1:** Patient eligibility criteria for DOORwaY90 trial

Inclusion criteria	Exclusion criteria
Willing, able, and mentally competent to provide written informed consent Age 18 or older at the time of consent Diagnosis of HCC with Liver Imaging Reporting and Data System (LI-RADS) 4 or 5 or by histology Treatment-naïve patients, including no prior locoregional therapies in the liver and no systemic therapy for HCC BCLC stage A, B1, B2, and C with maximal single tumor size of ≤ 8 cm and sum of the maximal tumor dimensions of ≤ 12 cm, with the entire tumor burden expected to be treatable within the perfused volume All tumors must be measurable by CT or MRI according to localized mRECIST At least one lesion ≥ 2 cm in diameter (long axis) measured according to mRECIST criteria by CT or MRI Child Pugh score A5 or A6 at baseline Albumin-Bilirubin (ALBI) grade = 1 or 2 at baseline Eastern Cooperative Oncology Group (ECOG) performance score 0 or 1 at baseline Adequate blood count, liver enzymes, and renal function at baseline Platelet count > 50,000/µl (no platelet transfusion or growth factors) White blood cell count ≥ 3 × 10^9^/l Hemoglobin >8.5 g/dl AST and ALT <5 × upper limit normal Bilirubin ≤ 2.0 mg/dl Albumin >3.0 g/dl Creatinine <2.0 mg/dl INR ≤ 2.0 Glomerular filtration rate > 50 Negative serum pregnancy test at baseline Life expectancy of ≥ 6 months with life expectancy of > 3 months if receiving no active treatment	Patient eligible for ablation or resection for their malignancy in the opinion of the investigator at screening visit Prior systemic anti-cancer therapy (including immunotherapy and/or targeted therapy), radiotherapy or use of other investigational agents for the treatment of HCC Intrahepatic arteriovenous shunting. (arteriovenous shunting resulting from a biopsy is allowed but must be embolized during the pre-treatment mapping procedure) Incompetent biliary duct system, prior biliary intervention or a compromised Ampulla of Vater Planned localized cancer treatment to the liver, other than the study treatment, during the study Planned systemic cancer treatment during the study Portal vein thrombosis Extrahepatic disease Patients with contraindications to angiography and selective visceral catheterization Evidence of extrahepatic collateral supply to the tumor Evidence of potential delivery of mean radiation dose > 30 Gy to the lungs (single treatment) Evidence of any detectable ^99m^Tc-MAA flow to outside of the liver in the abdomen, after application of established angiographic techniques to stop or mitigate such flow (e.g., placing catheter distal to gastric vessels or coiling) Evidence that < 33% of the total liver volume is disease-free and will be spared ^90^Y resin microsphere treatment Prior liver resection and/or liver transplant Female patients who are pregnant, breastfeeding, or premenopausal and unwilling to use an effective method of contraception through the 1-year follow-up; males unwilling to use effective method of contraception for 30 days post-procedure Medical history of clotting disorders Underlying pulmonary disease requiring chronic oxygen therapy Evidence of portal hypertension with ascites as seen on cross-sectional imaging or history of variceal bleeding within 6 months before screening Concurrently enrolled in another study unless it is an observational, non-interventional study Active infection (hepatitis B (HBV) infection with ongoing HBV treatment and successfully treated hepatitis C infection is allowed) History of other cancer with current active treatment Patients with drug or alcohol dependency (within 6 months of study entry) in the opinion of the investigator History of severe allergy or intolerance to contrast agents, narcotics, or sedatives Any condition that, in the opinion of the investigator, would interfere with safe delivery of the study treatment or with the interpretation of study results

### Treatment

#### Pre-SIRT

The methods of catheter angiography and hepatic arterial mapping for SIRT are well established and reviewed elsewhere [16]. Briefly, angiography is performed to identify the appropriate catheter position for administration of the microspheres and to visualize any hepatic or gastrointestinal vessels that may result in non-target dose deposition. If found, these vessels must be successfully circumvented before proceeding with SIRT, either by adjusting the catheter placement or by performing prophylactic embolization. After this evaluation, technetium-99m (^99m^Tc)-macroaggregated albumin (MAA) is administered via the catheter at the planned treatment position as a surrogate for the ^90^Y microspheres. The biodistribution of the ^99m^Tc-MAA is assessed via planar scintigraphy of the chest and abdomen and single photon emission computed tomography/computed tomography (SPECT/CT) of the liver.

Target and non-target tumor compartments and the treated non-tumoral liver tissue compartments are segmented, and volumes and ^99m^Tc-MAA uptake determined. The patient-specific target tumor-to-normal ratio (TNR) that is needed as input for the partition model is then calculated. The catheter tip during ^99m^Tc-MAA injection would typically be placed at the same anatomical position from which ^90^Y resin microspheres will be administered. The exception is when segmental treatments are planned, in which case, lobar ^99m^Tc-MAA injection may be performed, at the discretion of the clinician. When a segmental treatment (number of segments ≤ 2) is planned, the TNR value is then set to 1.0 (i.e., plan as a single compartment), and the minimum planned dose to the segment is > 150 Gy. Multi-vessel treatments are permitted. However, a cone-beam computed tomography (CBCT) or in-room fan beam CT should be performed to adequately determine the perfused volume. ^99m^Tc-MAA is administered at each location; exceptions include segmentectomy cases. Clinical justification for adjustment or alteration of catheter position between sessions is documented. All pre-SIRT images and contours are assessed jointly by the treatment physician and the Eligibility Review Committee to confirm treatment planning dosimetry and approach based on the dosimetry principles and constraints outlined next.

#### Treatment plan dosimetry

CBCT or in-room fan beam CT is used for volume calculation. 3D volumetric software is used to segment the total liver volume (TLV), the volume of perfused normal liver parenchyma (NLp) and the treated tumor (TT) compartments in the liver, and to determine their volume (in ml) and the total ^99m^Tc-MAA counts in the NLp and TT compartments. Soft-tissue density of 1.06 g/ml is used to determine the mass (in kg) of the TT compartment (M_tumor_) and the mass of the perfused normal liver compartment (M_NLp_). The patient-specific tumor uptake ratio, R, is calculated (using MIM Maestro) as:

- R = tumor compartment (total ^99m^Tc-MAA counts/total volume of interest)/perfused normal liver compartment (total ^99m^Tc-MAA counts/total volume of interest)

Based on partition model equations [17], the administered activity (A_admin_) required to meet the intended mean dose to the tumor compartment, D_tumor_, is calculated according to the formula:

- A_admin_ [GBq] = D_tumor_ [Gy] × M_tumor_ [kg]/(49.7 [Gy-kg/GBq] × (1-LSF) × R × M_tumor_/(M_NLp_ + R × M_tumor_)), where LSF is the lung shunt fraction.

The mean dose to the perfused normal liver compartment, D_NLp_, is calculated as:

- D_NLp_ [Gy] = D_tumor_ [Gy]/R.

#### Lung dosimetry

Regions of interest are drawn around the lung region and the liver region in both the anterior and the posterior views of planar scintigraphy. The view with the highest ^99m^Tc-MAA counts for the liver region is used to determine the ^99m^Tc-MAA counts in the lung region and the ^99m^Tc-MAA counts in the liver region for the calculation of the lung shunt fraction (LSF). The LSF is calculated using the following formula:

- LSF = (counts in lung region)/((counts in lung region) + (counts in liver region))

The mean lung dose (D_lung_) corresponding to a specific LSF can be determined as:

- D_lung_ [Gy] = A_admin_ [GBq] × LSF × 49.7 [Gy-kg/GBq]/M_lung_ [kg], where M_lung_ is the mass of lung tissue [kg].

The M_lung_ can be calculated using a patient-specific CT scan or can be estimated as 1 kg.

#### Treatment plan dosimetry constraints

**Normal liver constraints:** patients are excluded if < 33% of the total liver volume based on body surface area (TLV^BSA^) is tumor free, and therefore, left untreated. The TLV^BSA^ is calculated based on the method of Vauthey et al. [15] as:

- TLV^BSA^ [ml] = − 794.4 + 1267.3 × (height [cm] × weight [kg]/3600)^0.5^

If the ^90^Y treatment-spared liver volume is ≥ 33% but < 40% of the TLV^BSA^, the dose to the perfused normal liver parenchyma will be limited to < 150 Gy [14]. If the ^90^Y treatment-spared liver volume is ≥ 40% of the TLV^BSA^, there will be no upper limit to the dose allowed to the perfused normal liver parenchyma [18].

**Lung mean dose (D**_**lung**_**) constraints**: D_lung_ ≤ 30 Gy for a single treatment, and the cumulative lung mean dose < 50 Gy if the patient is re-treated.

**Tumor mean dose (D**_**tumor**_**) constraints**: the planned mean dose to the tumor must be in the range of 150–400 Gy. The lower limit of 150 Gy was based on the estimated mean dose required to achieve a complete response (CR) in previous studies [19, 20]. In the unlikely event that the planned mean dose to the total tumor compartment cannot exceed 150 Gy, the patient is considered a screening failure.

#### SIRT procedure

All treatments are performed with 3-day FLEXdose delivery option in a single treatment session using a selective or lobar approach. To optimize the predictive value of the ^99m^Tc-MAA intra-hepatic distribution, the catheter tip during ^99m^Tc-MAA injection is recommended to be placed at the same anatomical position from which ^90^Y resin microspheres will be administered (a deviation greater than ± 0.5 cm is allowed, if necessary, but must be documented; likewise, the occurrence of stasis will be documented).

#### After the SIRT procedure

Post-treatment assessment is conducted by imaging of the patient’s abdomen with ^90^Y SPECT/CT or ^90^Y PET/CT. Post-SIRT images are assessed by the Eligibility Review Committee using FDA-approved software to qualitatively confirm tumor targeting, quantify the mean tumor dose delivered, and to decide if re-treatment may be necessary.

#### Repeat SIRT

If the mean tumor dose delivered, based on post-treatment ^90^Y SPECT/CT or ^90^Y PET/CT imaging, was estimated to be < 100 Gy, SIRT re-treatment may be performed at the discretion of the treating physician within 4 weeks of the index procedure.

### Assessments

Treated patients will be assessed at baseline (no more than 28 days before SIRT), and at 1, 2, 4, 6, 9 and 12 months (± 2 weeks) and at 24 months (± 1 month) after SIRT. All patients are assessed according to the schedule summarized in Table 2. All images used to assess tumor response are reviewed at an independent centralized laboratory (American College of Radiology Center for Research and Innovation, Philadelphia, PA; ACR). Patients may be withdrawn from the study due to loss of follow-up, death, withdrawal of consent, adverse events, investigator decision, or if there is failed SIRT implantation. If a patient withdraws from the study a study completion electronic case report form must be completed, describing the reason for discontinuation. After 24 months and exit from the study, patients will be assessed and managed as per usual care.

**Table 2:** DOORwaY90 trial assessment schedule

Evaluations	Baseline^1^	Eligibility review committee approval	SIRT treatment	Re-treatment^2^	1 month (± 2 weeks)	Follow-up visits 2, 4, 6, 9 and 12 months (± 2 weeks) post-index procedure	2 years (± 1 month) clinic/phone
Patient informed consent	X						
Inclusion/exclusion assessment	X						
Physical exam including vital signs	X					X	
Demographics and medical history	X						
Pregnancy test	X						
Child–Pugh assessment	X			X			
Laboratory tests^3^	X		X	X	X	X	
^99m^Tc-MAA lung shunt scan and liver SPECT/CT	X						
Imaging (CT or MRI) of chest, abdomen, and pelvis	X					X^4^	
Hepatic angiography	X		X	X			
Treatment plan assessment	X						
ECOG performance test	X						
Medication assessment	X		X	X		X^5^	
Tumor response (mRECIST)^6^	X					X	
CBCT (or in-room fan-beam CT)	X		X	X			
^90^Y resin microsphere treatment			X	X			
Post-treatment imaging of abdomen with ^90^Y SPECT/CT or ^90^Y PET/CT			X	X			
Adverse event assessment	X		X	X	X	X	X
Quality of life questionnaires (EQ-5D-5L, FACT-Hep)			X^7^			X	

^1^Performed within 28 days prior to SIRT treatment procedure

^2^SIRT re-treatment may occur at the discretion of the treating physician within 4 weeks of index procedure

^3^CBC/Diff, biochemistry, total protein, AST, ALT, ALP, bilirubin, albumin, creatinine, prothrombin time, partial thromboplastin time, international normalized ratio. Hepatitis B and C, and serology at baseline visit only

^4^Modality of imaging (CT or MRI) must match method used at baseline

^5^Information on all concomitant medications collected for first 2 months; after 2 months, only oncologic and/or liver specific medications

^6^Images sent to core laboratory for analysis

^7^Completed pre-procedure

### Outcomes

The co-primary endpoints are localized objective response rate (ORR; CR or partial response [PR]) using modified Response Evaluation Criteria in Solid Tumors (mRECIST) [21] with best response through 9 months, and duration of response (DoR), defined as the time-interval from first achieving a response (i.e., CR or PR) until disease progression (≥ 6 months for ≥ 60% of responders).

Secondary endpoints include: grade ≥ 3 toxicity (Common Terminology Criteria for Adverse Events, CTCAE v5.0) at 3 months and 6 months; quality of life (QoL) measured by Functional Assessment of Cancer Therapy-Hepatobiliary (FACT-Hep) questionnaire and the five-level EuroQol five-dimensional questionnaire (EQ-5D-5L); liver resection rate; and liver transplant rate.

### Sample size calculation and statistical considerations

Continuous data will be summarized using descriptive statistics: sample size, mean, standard deviation, standard error, median, and range or interquartile range. Discrete variables will be summarized using frequency counts and percentages. All statistical analyses will be performed using SAS (version 9.4 or higher, SAS Institute Inc. Cary, NC), R (version 3.2 or higher, R Foundation for Statistical Computing, Vienna, Austria) or other widely accepted statistical or graphical software.

Primary analyses will be conducted under the principles of intent-to-treat (ITT), using the full analysis set (FAS) as defined in ICH E9 (Statistical Principles for Clinical Trials). Additionally, a per-protocol (PP) population is defined, including ITT patients who met all inclusion and exclusion criteria, received the intended dose of 150–400 Gy, and had no major protocol violations.

Methods for replacing missing data such as last value carried forward or multiple imputation will not be routinely used for the primary study analyses. For the purposes of defining the primary endpoints, death before the first attempt at evaluation will be defined as failure of the endpoint in question, and if no evaluable mRECIST data are collected for a patient, they will be classified as a failure for the ORR endpoint and as missing data for the DoR endpoint. Planned subgroup analyses will be conducted to evaluate consistency of results by gender and dosimetry method. Additionally, a dose–response analysis will be conducted to estimate the threshold mean tumor dose for response for tumors ≥ 3 cm. For the co-primary endpoint ORR, all lesions in the treated portion of the liver will be included for analysis, with up to 2 lesions per patient defined as target lesions and the rest non-target lesions. ORR results will be presented as the proportion of successes (CR or PR) with corresponding confidence limits calculated by the exact binomial (Clopper-Pearson) method. The performance goal is a lower confidence interval for best response on ORR of 40%. A DoR of ≥ 6 months in ≥ 60% of responding patients is the goal of the co-primary endpoint.

An overall summary of adverse events (AEs) will be presented, which will include the number and percentage of patients with at least one AE, and the total number of AEs. All AEs will be adjudicated by a Clinical Events Committee (CEC). AEs will additionally be classified according to the Medical Dictionary for Regulatory Activities (MedDRA) dictionary, summarized by severity, relationship to SIRT, outcome and seriousness. Serious adverse events (SAEs) will be summarized according to severity and relationship. Multiple occurrences of the same AE are counted once at the maximum grade and strongest relationship, as appropriate.

The postulated success rate for the ORR primary endpoint is estimated to be 55–60% based on previous experience with the study device and expert opinion on its effectiveness in the target population; as stated, the performance goal applied to the lower confidence bound for this estimate is 40%. Given a two-sided test at an alpha of 0.05 and desired power of 80%, the required evaluable sample size using the exact method for a single proportion and the worst estimate above is no greater than 90. Therefore, a study sample size of 100 is targeted to provide adequate powering for hypothesis testing as described above. Consent from up to 150 patients is anticipated to achieve the sample size of 100 patients, based on previous experience of eligibility failure of 33%. The overall study duration, from screening the first patient to the final follow-up visit, data analysis and final report, is expected to be approximately 39 months.

## Discussion

The DOORwaY90 trial will provide clinically relevant and novel information on the effectiveness and safety of ^90^Y resin microspheres for the treatment of unresectable HCC when personalized dosimetry methods are used. Previous large trials of SIRT with ^90^Y resin microspheres have not used personalized dosimetry [11–13], and subsequent analyses have suggested that sub-optimal doses have been used in these trials, resulting in sub-optimal effects of SIRT [19, 22]. A recent trial using personalized dosimetry with ^90^Y glass microspheres further emphasized the need for a personalized approach to SIRT dosimetry to achieve optimum results [23]. However, because of differences in the size, distribution, and specific activity of glass and resin microspheres, dosimetry findings cannot be translated from one product to the other, and therefore, the DOORwaY90 trial is needed to provide information on the impact of personalized dosimetry on the efficacy of ^90^Y resin microspheres.

The delivered mean tumor dose of < 100 Gy to trigger possible SIRT re-treatment is 66% of the minimum target tumor dose. This cut-off was used partly because there is an error of at least 20% in quantifying post-SIRT ^90^Y SPECT/CT or ^90^Y PET/CT-based tumor doses [24]. Furthermore, an average tumor dose of 110 Gy has been shown to result in tumor response in > 50% of patients (according to RECIST; and according to EASL guidelines, response was achieved in > 70% of patients) [20]. Therefore, the mean tumor dose cut-off of 100 Gy should avoid unnecessary SIRT re-treatment.

There is increasing evidence that SIRT may have the potential to downstage unresectable HCC, or act as a bridge to transplantation. This needs investigation in dedicated clinical studies, but the DOORwaY90 trial includes patients with earlier stage HCC than in previous prospective trials of ^90^Y resin microspheres and may help provide further insight into the ability of SIRT to convert patients to resectability. The DOORwaY90 trial further offers the opportunity to assess the safety profile of ^90^Y resin microspheres in the US population—an important consideration as many systemic treatments for HCC have safety profiles that can adversely impact patient QoL.

The DOORwaY90 study design is unique for several reasons. The DOORwaY90 study is the first prospective, multicenter US trial to utilize personalized ^90^Y dosimetry with ^90^Y resin microspheres: the use of a patient-specific target TNR allows a personalized prescribed activity that results in a dose that achieves maximum damage to the tumor, with minimal damage to healthy tissue. It is also the first study to employ an Eligibility Review Committee to determine patients’ treatment plans. In addition, unlike previous studies, tumor responses will be evaluated by an independent review body (ACR), and using novel imaging software (MIM Sureplan) we will assess if tumors achieve a minimum threshold dose to determine if patients are eligible for early retreatment. This study also utilizes a well-controlled patient population with a well-defined safety margin. The FLEXdose delivery program for ^90^Y resin microspheres ensures that all sites are using the same activity per sphere, that increases confidence in the entire planned activity being delivered without the potential for unplanned stasis (the 3-day FLEXdose delivery option also reduces further any risk of a macroembolic effect). The co-primary endpoints of ORR and DoR used in the DOORwaY90 trial were chosen as a measure of local disease control. As SIRT is a locoregional therapy, assessment of local disease control is a more relevant measure of effectiveness than overall survival.

The results of the DOORwaY90 trial will help refine the potential benefits of SIRT with ^90^Y resin microspheres as first-line therapy for HCC and may strengthen future consensus guidelines. If the DOORwaY90 trial meets its co-primary endpoints, personalized SIRT may become a new standard of care for patients with unresectable HCC. Such a treatment has the potential to facilitate liver resection or transplantation, and a consequent increased life expectancy and enhanced QoL for these patients.

## Supplementary Information

**Additional file 1.** All relevant institutional and local ethics committee approvals were gained before commencing the study.

## Data Availability

Not applicable for this publication. In future, it is our intention that results will be fully disclosed in ClinicalTrials.gov, by presentation at international congresses and via publication in a peer-reviewed journal.

## Abbreviations

AEs: Adverse events
BCLC: Barcelona Clinic Liver Cancer
CBCT: Cone-beam computed tomography
CEC: Clinical Events Committee
CR: Complete response
CRO: Contract research organization
CT: Computed tomography
CTCAE: Common terminology criteria for adverse events
DoR: Duration of response
DSMB: Data and Safety Monitoring Board
EASL: The European Association for the Study of the Liver
EQ-5D-5L: Five-level EuroQol five-dimensional questionnaire
FACT-Hep: Functional Assessment of Cancer Therapy-Hepatobiliary
HCC: Hepatocellular carcinoma
IRB: Institutional Review Board
ITT: Intent to treat
LSF: Lung shunt fraction
MedDRA: Medical Dictionary for Regulatory Activities
MAA: Macroaggregated albumin
ORR: Objective response rate
PP: Per protocol
PR: Partial response
QoL: Quality of life
mRECIST: Modified Response Evaluation Criteria in Solid Tumors
SAEs: Serious adverse events
SIRT: Selective internal radiation therapy
SPECT: Single photon emission computerized tomography
TLV^BSA^: Total liver based on body surface area
TNR: Tumor-to-normal ratio
TT: Treated tumor
