# Supplementary material for: A prospective, multicenter, open-label, single-arm clinical trial design to evaluate the safety and efficacy of 90Y resin microspheres for the treatment of unresectable HCC: the DOORwaY90 (Duration Of Objective Response with arterial Ytrrium-90) study
Source: BMC Gastroenterol. 2022 Mar 28;22:151. doi: 10.1186/s12876-022-02204-1 (PMC8962126; doi:10.1186/s12876-022-02204-1)
Supplement: Supplementary file 1 — Additional file 1. All relevant institutional and local ethics committee approvals were gained before commencing the study. [file 12876_2022_2204_MOESM1_ESM.docx]

Supplementary file to:

**A prospective, multicenter, open-label, single-arm clinical trial design to evaluate the safety and efficacy of ^90^Y resin microspheres for the treatment of unresectable HCC: the DOORwaY90 (Duration Of Objective Response with arterial Ytrrium-90) study**

Armeen Mahvash, Steven Chartier, Mark Turco, Paula Habib, Steven Griffith, Scott Brown, S. Cheenu Kappadath

List of participating sites and Institutional Review Board approvals

| **Site Name** | **Institutional Review Board** | **Reference Number** |
| --- | --- | --- |
| Inland Imaging | WCG IRB  1019 39th Ave SE, Suite 120  Puyallup WA 98374 | Study number: 1304433  IRB tracking number: 20210253 |
| Beth Israel Deaconess Medical Center | Dana-Farber Cancer Institute  450 Brookline Avenue, OS229  Boston, MA 02215 | 407665 |
| The Cleveland Clinic Foundation | Cleveland Clinic IRB OS-1 Cleveland Clinic 9500 Euclid Avenue Cleveland, OH 44195 | 21-1179 |
| Clinical Research Institute at Methodist Health System | WCG IRB  1019 39^th^ Ave SE, Suite 120  Puyallup WA 98374 | Study number: 1315036  IRB tracking number: 20210253 |
| Emory University School of Medicine | Emory University IRB  201 Dowman Drive  Atlanta, Georgia 30322 | STUDY00002540 |
| Hospital of the University of Pennsylvania | University of Pennsylvania Institutional Review Board  3600 Civic Center Blvd.  9th Floor Philadelphia, PA 19104 | 849177 |
| Massachusetts General Hospital | Dana-Farber Cancer Institute  450 Brookline Avenue, OS229  Boston, MA 02215 | 407665 |
| Miami Cardiac and Vascular Institute at Baptist Hospital | WCG IRB  1019 39^th^ Ave SE, Suite 120  Puyallup WA 98374 | Study number: 1319065  IRB tracking number: 20210253 |
| Stanford University | Stanford University  Stanford, CA 94305 [Mail Code 5579] | 62236 |
| University of Kansas Medical Center | WCG IRB  1019 39^th^ Ave SE, Suite 120  Puyallup WA 98374 | Study number: 1319218  IRB tracking number: 20210253 |
| University of Minnesota | University of Minnesota  McNamara Alumni Center, Room 350-2  200 Oak Street S.E.  Minneapolis MN 55455 | STUDY00013092 |
| University of Texas Health Science Center at Houston (UTHealth) | UTHealth  6410 Fannin Street, Suite 1100  Houston TX 77030 | HSC-MS-21-0124 |
| UT MD Anderson Cancer Center | MD Anderson Office of Human Subject Protection 7007 Bertner Ave – Unit 1637 Houston, TX 77030 | 2021-0171_MOD001 |
| Northwestern University | Northwestern University  750 N Lake Shore Drive  Rubloff Building, 7th Floor | STU00215321 |
| Providence Holy Cross Medical Center | WCG IRB  1019 39th Ave SE, Suite 120  Puyallup WA 98374 | Pending |
| Wake Forest University | Wake Forest IRB  Medical Center Boulevard  Winston-Salem, NC 27157 | IRB00079129 |
